# Effectiveness of Spine Correction Belt for Treatment of Diaphragmatic Flutter

**DOI:** 10.5334/tohm.967

**Published:** 2025-02-14

**Authors:** Yoshiaki Kazama, Yuichi Ando, Masashi Suzuki, Juichi Sato

**Affiliations:** 1 Department of General Medicine, Nagoya University Hospital, 65 Tsurumai-cho, Showa-ku, Nagoya 466–8560, Japan; 2 Department of Clinical Laboratory, Nagoya University Hospital, 65 Tsurumai-cho, Showa-ku, Nagoya 466–8560, Japan

**Keywords:** Diaphragmatic flutter, posture, spine correction belt

## Abstract

**Background:**

Diaphragmatic flutter is an unusual movement disorder characterized by involuntary and repetitive contractions of the diaphragm. The pathophysiology is unclear. Its treatment is very difficult and challenging.

**Case report:**

A 70-year-old man presented with diaphragmatic flutter associated with severe abdominal pain in a sitting position, which stopped in a supine position. Videofluoroscopy clearly depicted diaphragmatic movements on postural change. A spine correction belt was effective for stopping diaphragmatic flutter.

**Discussion:**

This is the first reported case of diaphragmatic flutter for which a spine correction belt was used as successful and safe treatment.

**Highlights:**

Diaphragmatic flutter is an unusual movement disorder of the diaphragm. We describe a 70-year-old man who presented with diaphragmatic flutter associated with severe pain in a sitting position, which stopped in a supine position. A spine correction belt was used as successful and safe treatment for diaphragmatic flutter.

## Introduction

Diaphragmatic flutter (DF) is an unusual movement disorder characterized by involuntary and repetitive contractions of the diaphragm [1,2]. While various causes may be associated with DF, most cases are idiopathic [3,4]. The exact mechanisms causing the diaphragm to flutter are still unclear. We report the case of a 70-year-old man who presented with DF associated with severe pain in a sitting position, which stopped in a supine position. Gradually raising his upper body up from a supine position induced the movement disorder, which was stopped by posture correction involving stretching the spine. This is the first reported case of DF for which a spine correction belt (SCB) was used as successful and safe treatment.

## Case Report

**Figure 1:**
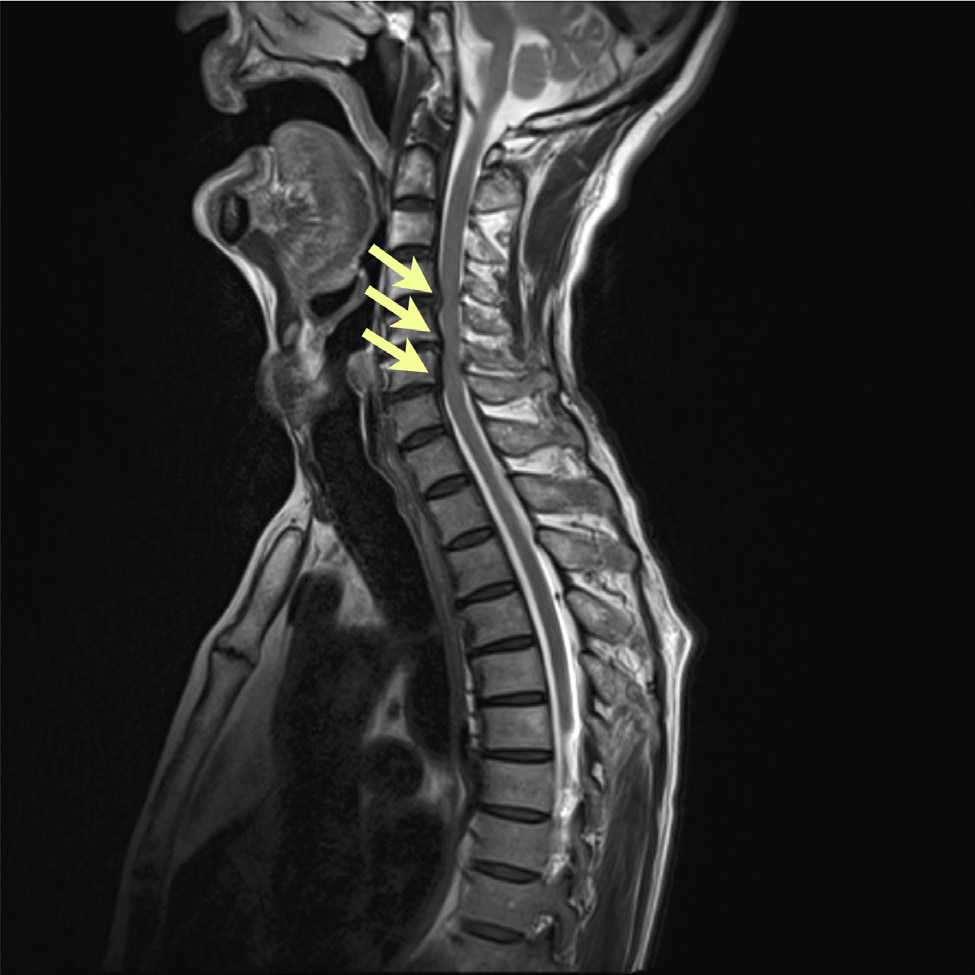
**MRI Sagittal image of the spine**. T2-weighted MRI shows mild cervical stenosis at C4–5, C5–6, and C6–7 levels (arrows).

In February 2019, a 70-year-old man felt a sudden stabbing pain in the right upper abdominal wall, and noticed repetitive rhythmic involuntary contractions of the wall. He had undergone laparoscopic cholecystectomy in April 2014. In May 2020, right anterior cutaneous nerve neurectomy was performed based on a diagnosis of anterior cutaneous nerve entrapment syndrome, but the symptoms did not improve. In September 2021, he was referred to our out-patient clinic, presenting with abnormal contractive movements of the anterior abdominal wall with severe pain. They were observed in a sitting position, but disappeared in a supine position (Video 1, Segments 1 and 2). The patient’s wife confirmed that the symptoms ceased during sleep. No abnormal movements were observed from a supine position to ~50 degrees (Video 1, Segment 3). Raising the body up further induced the movements immediately (Video 1, Segment 4). The abdominal pain disappeared along with disappearance of abnormal movements. He did not feel dyspnea, presumably due to normal breathing movement superimposed by DF and/or compensation by other respiratory muscles. Deep breaths and coughing did not induce DF. The movements could not be suppressed by breath-holding or deep inspiration. No palatal tremor was present and there were no other abnormal neurological signs. Routine blood test results were all normal. Findings from brain computed tomography and magnetic resonance imaging (MRI) revealed nothing of note. Spinal MRI showed mild cervical stenosis with C4–C5, C5–C6, and C6–C7 disc herniations and thickening of the posterior longitudinal and yellow ligaments (Figure 1). No neural foraminal stenosis was observed at C4–C5, C5–C6, or C6–C7 levels.

**Video 1:** **Abnormal movements of the abdominal wall**. Abnormal repetitive contractions of the abdominal wall were observed on changing the position and posture. A spine correction belt stopped DF (Seg. 8).

**Figure 2:**
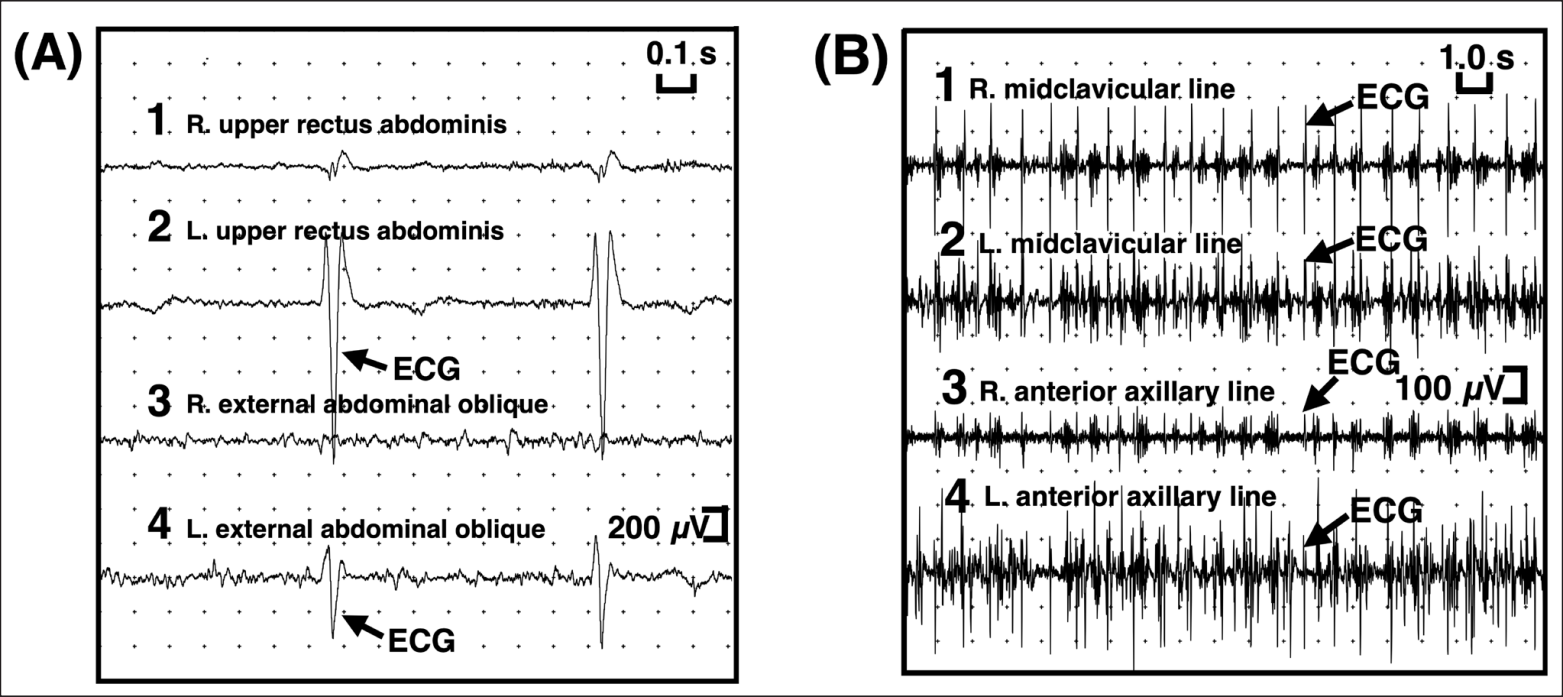
**Surface EMG in a sitting position**. EMG electrodes placed (A) over the rectus abdominis and external abdominal oblique muscles, and (B) at costal margins along the midclavicular line and anterior axillary line. ECG, electrocardiogram. R, right. L, left.

In order to distinguish diaphragm fluttering from contractions of abdominal muscles, fluoroscopy was performed, demonstrating real-time movements of the diaphragm, and gastric and intestinal air masses. Videofluoroscopy of the screen was continuously recorded from a supine to sitting position by gradually raising the X-ray fluoroscopic table (Video 2, Segments 9~13). Normal movements of the diaphragm were observed in a supine position (Video 2, Segment 9) and from a supine position to ~50 degrees (Video 2, Segment 10). The X-ray fluoroscopic table was stopped temporarily in the position (approximately 50 degrees) where repetitive irregular flutters of both hemidiaphragms were observed (Video 2, Segment 11). High-frequency flutter of the diaphragm was observed by further raising the X-ray fluoroscopic table (Video 2, Segment 12) to a sitting position (Video 2, Segment 13) at a rate of approximately 70 contractions per minute, predominantly involving the left hemidiaphragm. Surface electromyography (EMG) in a sitting position was performed involving several abdominal muscles on both sides. No significant EMG activities were observed involving the rectus abdominis or external abdominal oblique muscles (Figure 2A). However, EMG activities responsible for abdominal wall movements were recorded bilaterally at costal margins along the midclavicular line and anterior axillary line (Figure 2B). The EMG activities were rhythmic, at approximately 1.2 Hz, but were not synchronous with QRS of the electrocardiogram (ECG), suggesting that the abdominal wall movements originated by active contraction of the diaphragm. Taking these findings into consideration, the abnormal movements were considered to be DF. Clonazepam, carbamazepine, diazepam, phenytoin, and haloperidol were ineffective. Intercostal nerve blocks were also ineffective. Our patient did not consent to any other invasive treatments.

**Video 2:** **Videofluoroscopy of diaphragm movements on changing the position and posture**. Videofluoroscopy of the screen was continuously recorded from a supine to sitting position by gradually raising the X-ray fluoroscopic table.

At one year and 8 months after he had initially visited our clinic, we noticed that posture correction including moving the head forward by stretching the spine stopped the abnormal movements (Video 1, Segments 5, 6, and 7). Therefore, an elastic SCB was introduced with the aim of straightening the spine and improving postural alignment, resulting in the complete resolution of DF without abdominal pain (Video 1, Segment 8). It was hard to stop abnormal movements by compressing and tightening abdominal muscles with a belt without posture correction. SCB was not a sensory trick for stopping DF. The patient was advised to use SCB while standing or walking, but to remove it during the night or in a supine position. There were no adverse or unanticipated events. The improvement of DF was observed over a 6-month follow-up period.

## Discussion

A diagnosis of DF is often difficult and delayed due to its rarity, variable clinical symptoms, and various underlying etiologies [3,4,5]. The literature shows that the terms of DF and belly dancer’s dyskinesia (BDD) are often used indistinctly and interchangeably. Walton, *et al*. [6] indicated that DF should be distinguished from BDD, since DF reflects abnormal activity of the diaphragm, and abdominal wall movements in BDD occur through contractions of the recti and oblique abdominal muscles. Thus, to distinguish between diaphragm and abdominal muscles contractions, fluoroscopy should be performed to confirm real-time diaphragm movement in combination with EMG. However, needle EMG of the diaphragm is sometimes uncomfortable for a patient and requires skilled techniques. Ultrasound imaging is also used for this purpose [7]. Two main mechanisms whereby the diaphragm flutters have been proposed [3,6,8]: (i) abnormal excitation of the phrenic nerve, either by the central nervous system or presence of irritating factors anywhere along the phrenic pathway, and (ii) irritation of the diaphragm.

Our case highlighted two important clinical implications. First, the abnormal movements of the diaphragm were related to a change in the position and posture. We considered that raising the body up from a supine position may have applied a load on the cervical spinal cord and/or nerve roots, as well as on the phrenic nerves. This pressure may have induced abnormal excitability of the phrenic pathway. Thus, attenuation and release of the compression effect with posture correction by stretching the spine may have stopped the abnormal movement. Videofluoroscopy clearly demonstrated the changing process of diaphragm movements from supine to sitting positions. These findings suggest that DF in our patient may not have been of cerebral origin, but of spinal origin. Whether the mild cervical stenosis was related to abnormal hyperexcitability of the phrenic nerve pathway remains unclear at present. While the exact pathophysiology in which the left hemidiaphragm was predominant in intensity of diaphragm contraction was not clarified, it may be due to different structures of the left and right phrenic nerves at the root of the neck, in the thorax, or in the mediastinum [9]. In addition, variation of the phrenic nerves and the presence of an accessory phrenic nerve may be taken into consideration.

Regarding the influence of postural change on involuntary abnormal movements, a similar phenomenon was reported involving dancing dorsal quadrilaterals (DDQ), a peripherally induced movement disorder that predominantly affects the dorsal quadrilateral muscles (trapezius and rhomboids) after upper spinal instrumentation [10]. The movements of DDQ occur when sitting or standing, and disappear when supine or during sleep. Interestingly, the abnormal movements reportedly disappeared by stretching the spine and correcting the posture by holding up the arms, as in our case. Partial but persistent nerve damage after spinal instrumentation, especially when complicated with hardware instability, might play a critical role in hyperkinetic movements. In our case, it is unclear at present whether partial injury of the abdominal cutaneous nerve or phrenic nerves after laparoscopic cholecystectomy occurred, leading to hyperexcitability of the phrenic pathway. DF was initially considered as a variant of palatal myoclonus, suggesting brainstem involvement, particularly the dentate-rubro-olivary pathway [1,6]. DF in our case was unlikely to be related to palatal myoclonus. Furthermore, the other condition with which DF may be confused is spinal myoclonus [11]. Myoclonus is defined as a sudden, shock-like involuntary movement due to muscle contraction or inhibition [12]. Kono, *et al*. [13] reported a case of idiopathic spinal myoclonus involving the abdominal wall and paraspinal muscles, which was considered to be different from BDD. Inghilleri, *et al*. [14] also reported a case of spinal myoclonus resembling BDD, possibly due to mild D10 disk herniation. Neither report provided any comments on diaphragm movements. Considering this differential diagnosis and involvement of only the diaphragm, our case supports the distinction of DF as a specific entity.

Second, SCB may be useful in the management of DF, presumably due to the reduction of pressure to the cervical spinal cord/and roots, as well as to the phrenic nerves. While several pharmaceuticals for DF such as antiepileptic and antipsychotic medications have been shown to be effective [4], most patients are refractory to pharmacologic treatments. Multiple pharmacologic trials failed in our patient. Some invasive treatments were reported to be effective [6], such as phrenic nerve block [5], phrenic nerve ablation [15], botulinum toxin A injection into the diaphragm [16], and resting the diaphragm long-term [17]. However, these invasive treatments require skilled techniques and appropriate facilities. In addition, some side effects including a paralyzed hemidiaphragm and decrease in lung volumes have been noted, and complete or partial success is not guaranteed in all patients. Furthermore, the recurrence of symptoms has been reported, and the long-term benefits remain unknown. In summary, our case clearly showed the influence of postural change on DF, and highlighted the importance of stretching the spine and stabilizing postural alignment to stop it. On the basis of these findings, SCB was introduced as effective and noninvasive treatment for DF. In this regard, it is interesting that Kobayashi, *et al*. [18] reported the usefulness of a soft neck brace in a patient who developed spinal segmental myoclonus after undergoing laminoplasty for spondylosis. The involuntary myoclonic jerky movements in the upper trapezius occurred only in sitting and standing positions, as in our case. The myoclonus was refractory to any medication, and intramuscular injection of botulinum toxin A into the upper back for 15 years since the onset of spinal segmental myoclonus. To our knowledge, this is the first reported case of DF for which SCB was used as successful and safe treatment without adverse effects. A trial of such a simple approach in patients with DF is warranted.

## Data Accessibility Statement

The data that support the findings of this study are available on request from the corresponding author.
